# Sex-Specific Consequences of Neonatal Stress on Cardio-Respiratory Inhibition Following Laryngeal Stimulation in Rat Pups

**DOI:** 10.1523/ENEURO.0393-17.2017

**Published:** 2018-01-04

**Authors:** Cécile Baldy, Simon Chamberland, Stéphanie Fournier, Richard Kinkead

**Affiliations:** 1 Department of Pediatrics, Centre de Recherche de l’Institut de Cardiologie et Pneumologie de Québec, Université Laval, Québec, G1V 4G5, Canada; 2 Department of Psychiatry and Neuroscience, Québec Mental Health Institute, Université Laval, Québec, G1J 2G3, Canada

**Keywords:** control of breathing, development, hormones, neonatal programming, sex-based differences, stress

## Abstract

The presence of liquid near the larynx of immature mammals triggers prolonged apneas with significant O_2_ desaturations and bradycardias. When excessive, this reflex (the laryngeal chemoreflex; LCR) can be fatal. Our understanding of the origins of abnormal LCR are limited; however, perinatal stress and male sex are risk factors for cardio-respiratory failure in infants. Because exposure to stress during early life has deleterious and sex-specific consequences on brain development it is plausible that respiratory reflexes are vulnerable to neuroendocrine dysfunction. To address this issue, we tested the hypothesis that neonatal maternal separation (NMS) is sufficient to exacerbate LCR-induced cardio-respiratory inhibition in anesthetized rat pups. Stressed pups were separated from their mother 3 h/d from postnatal days 3 to 12. At P14–P15, pups were instrumented to monitor breathing, O_2_ saturation (S*p*o_2_), and heart rate. The LCR was activated by water injections near the larynx (10 µl). LCR-induced apneas were longer in stressed pups than controls; O_2_ desaturations and bradycardias were more profound, especially in males. NMS increased the frequency and amplitude of spontaneous EPSCs (sEPSCs) in the dorsal motor nucleus of the vagus (DMNV) of males but not females. The positive relationship between corticosterone and testosterone observed in stressed pups (males only) suggests that disruption of neuroendocrine function by stress is key to sex-based differences in abnormal LCR. Because testosterone application onto medullary slices augments EPSC amplitude only in males, we propose that testosterone-mediated enhancement of synaptic connectivity within the DMNV contributes to the male bias in cardio-respiratory inhibition following LCR activation in stressed pups.

## Significance Statement

Neuroendocrine signals are essential to brain development, including sexual differentiation. Perinatal stress and male sex are significant risk factors for cardio-respiratory failure in infants. Here, we show that the neuroendocrine disruption induced by neonatal maternal separation (NMS) is sufficient to exacerbate reflexive cardio-respiratory inhibition in rat pups, especially in males. Electrophysiological recordings indicate that stress augments excitatory currents converging onto the dorsal motor nucleus of the vagus (DMNV), a brainstem region that inhibits heart rate and in which enhanced synaptic elements have been reported in victims of sudden infant death. Dysregulation of testosterone secretion is a plausible explanation for these abnormalities. These results provide new insights into the origins of sex-based differences in neurologic dysfunction, including cardio-respiratory failure in infants.

## Introduction

Recurrent apneas with O_2_ desaturations and bradycardias are an important cause of hospitalization and morbidity in infants, especially those born preterm. In addition to inadequate responsiveness to respiratory stimuli (O_2_ and CO_2_), the inability to coordinate breathing with swallowing contributes to this problem (Martin and Wilson, 2012). During feeding or esophageal reflux, the presence of liquids near the larynx triggers the laryngeal chemoreflex (LCR), a set of responses aiming to prevent aspiration of foreign substances in the airways. Owing to neural control immaturity, infants are unable to cough or swallow and LCR activation results in prolonged apneas with profound O_2_ desaturations and bradycardias that can be life threatening. Consequently, it was hypothesized that excessive cardio-respiratory inhibition resulting from abnormal or delayed LCR maturation contributes to sudden infant death syndrome (SIDS; Kinney et al., 2009; Praud, 2010). Despite its significant impact on infant health, our understanding of the factors interfering with LCR development and function remains limited. However, the SIDS literature identifies male sex and exposure to systemic (e.g., nicotine) and environmental stressors (e.g., socioeconomic status) as significant risk factors for cardio-respiratory failure (Kinney and Thach, 2009).

Neonatal stress affects brain development in a sex-specific manner and predisposes to various neurologic disorders (Shonkoff et al., 2009; Bale and Epperson, 2015). Because perinatal stress interferes with androgen secretion in newborn (Weisz and Ward, 1980; Fournier et al., 2013) and testosterone contributes to brain masculinization by inducing or repressing synapse formation in a region-specific manner during early life (Schwarz and McCarthy, 2008; Lenz et al., 2012), it is therefore plausible that the neuroendocrine disruption resulting from adverse environments is sufficient to interfere with cardio-respiratory control development in newborn and explain the male bias in dysfunction. The higher testosterone levels reported in infant victims of unexpected, unexplained death are consistent with this hypothesis (Emery et al., 2005). However, the confounding factors related to the environment and maternal lifestyle make it difficult to address this issue and the conclusions that can be drawn from clinical studies remain limited.

With that in mind, we hypothesized that neonatal stress, in the form of neonatal maternal separation (NMS), aggravates the cardio-respiratory inhibition triggered by LCR stimulation in rat pups. Besides being a clinically relevant model of neonatal stress (Doom and Gunnar, 2013; Battaglia et al., 2014), NMS offers the possibility to evaluate the impact of stress-related neuroendocrine disruption on respiratory function in newborn without confounding factors related to the presence of drugs, nicotine, and/or other uncontrolled environmental conditions. Moreover, the sex-specific effects of NMS also make it possible to better understand the origins of sex-based differences in the manifestation of respiratory disorders in newborn.

To address this issue, we used an established newborn pup preparation (Xia et al., 2008) combined with pulse oximetry to first compare the cardio-respiratory responses to repeated LCR stimulation between pups subjected to NMS (3 h/d, postnatal days 3-12) versus controls (undisturbed). To gain mechanistic insight, we then performed single cell recordings of spontaneous and miniature EPSCs (mEPSCs) to evaluate the impact of NMS and sex on synaptic activity in the commissural region of the nucleus of the tractus solitarius (NTScom) and the dorsal motor nucleus of the vagus (DMNV). In addition to being one of several sites of central CO_2_ chemoreception in the central nervous system (Dean and Putnam, 2010), these caudo-dorsal medullary regions respectively receive sensory afferents from the laryngeal receptors and send parasympathetic projections to numerous viscera, including the heart and airways (Nosaka et al., 1979; Haxhiu and Loewy, 1996; Mendelowitz, 1999; Jordan, 2001; Machhada et al., 2015). Based on the sex-specific effects of stress on cardio-respiratory responses and synaptic function within the DMNV, the effect of preincubating slices with testosterone before EPSC recording was measured in these neurons.

## Materials and Methods

### Ethical approval and compliance

Laval University Animal Care Committee approved all the experimental procedures described in this article (CPAC protocol #2013015-1); the protocols were in accordance with the guidelines detailed by the Canadian Council on Animal Care. All personnel involved in this research followed appropriate training and complied with institutional and national ethical guidelines.

### Origin and source of the animals

Experiments were performed on Sprague Dawley male and female rat pups. Details of animal distribution among groups and sex is provided in the results section for each experimental series. All animals were born and raised in our animal care facilities. Dams and males used for mating were obtained from Charles River Canada.

### Access to food and water

Rats were supplied with food and water ad libitum and maintained in standard laboratory and animal care conditions (21°C, 12/12 h light/dark cycle; lights on at 07:00 A.M. and off at 07:00 P.M.). Experiments were performed on 14- to 15-d-old rat pups, 2 d after completion of the NMS protocol.

### Mating procedures and neonatal stress protocol

Virgin females were mated and delivered 10–15 pups. Two days after delivery, litters were culled to 12 pups, when necessary, with a roughly equal number of males and females. The NMS protocol was identical to the one used in previous studies (Genest et al., 2004; Fournier et al., 2015). Briefly, the entire litter was separated from their mother for 3 h/d (09.00-12.00 h) from postnatal days 3 to 12. Separated pups were placed in a temperature (35°C)- and humidity (45%)-controlled incubator and isolated from each other by an acrylic partition. This temperature was chosen because it is within the thermoneutral range for rat pups of this age (Mortola, 2001). Data obtained from this experimental group were compared with that of animals in which the nest was not disturbed and therefore not subjected to the NMS procedure. As discussed previously (Gulemetova and Kinkead, 2011), these animals are the most desirable control group for investigations of the effects of maternal separation (Lehmann and Feldon, 2000). In each series of experiments, each group of rats was composed of animals originating from multiple litters to avoid litter-specific effects.

### LCR protocol and rationale for analysis

This procedure was based on a protocol developed previously (Xia et al., 2008). Briefly, each pup was anesthetized with urethane (1 mg/kg) and chloralose (20 mg/kg) by intraperitoneal injection. Once a surgical plane of anesthesia was reached (∼20 min), the animal was placed in the supine position, a small thermistor probe was inserted into the rectum to record body temperature, which was controlled at 35°C with a homeothermic blanket (Harvard Appratus). O_2_ saturation (S*p* o_2_) and heart rate were measured with pulse oxymetry (Mouse Ox, Starr Life Sciences) by placing a sensor onto the thigh of the pup. Hooked silver wires (diameter of 0.25 mm; Precision Instrument Inc) were introduced into the intercostal muscles in the lowest part of the rib cage to record electromyography (EMG) as an index of respiratory activity. A grounding wire was inserted subcutaneously into the skin over the abdomen. The EMG signal was amplified, filtered (A-M Systems, model 1800), and recorded with a data acquisition system (Windaq, DataQ Instrument). A midline skin incision was then made in the neck and the cervical trachea was freed from adjacent tissues. The recurrent and superior laryngeal nerves were identified and carefully avoided during the dissection. The trachea was cut partially with a transverse incision so that the lumen was visible. A polyethylene tubing (PE-50, Clay Adams, Beckton Dickinson) was inserted slowly into the rostral part of the trachea until the tip reached the larynx. The pup’s head was then tilted backward slightly to facilitate breathing via the opened trachea and ensure that the water injected near the larynx would drain via the nose and mouth and not be aspired into the lower airways and lungs.

**Figure 1.**
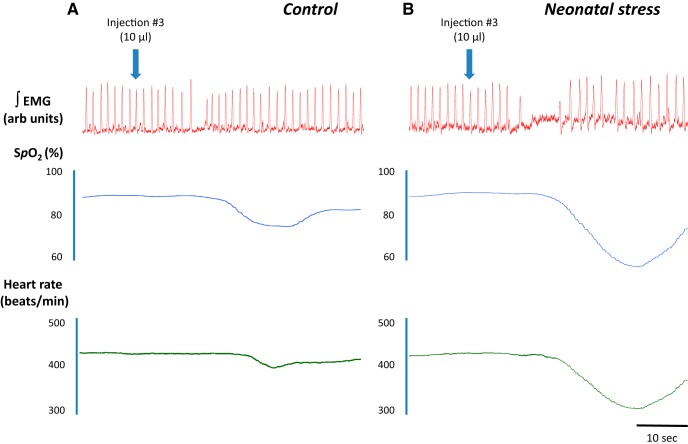
Original recordings comparing cardio-respiratory responses to the third LCR activation by intratracheal water injection (10 µl) between pups subjected to (***A***) control conditions and (***B***) neonatal stress in the form of maternal separation. The traces illustrate (from top to bottom): intercostal EMG, S*p* o_2_, and heart rate. These recordings were obtained following the third injection in 15-d-old male pups.

**Figure 2.**
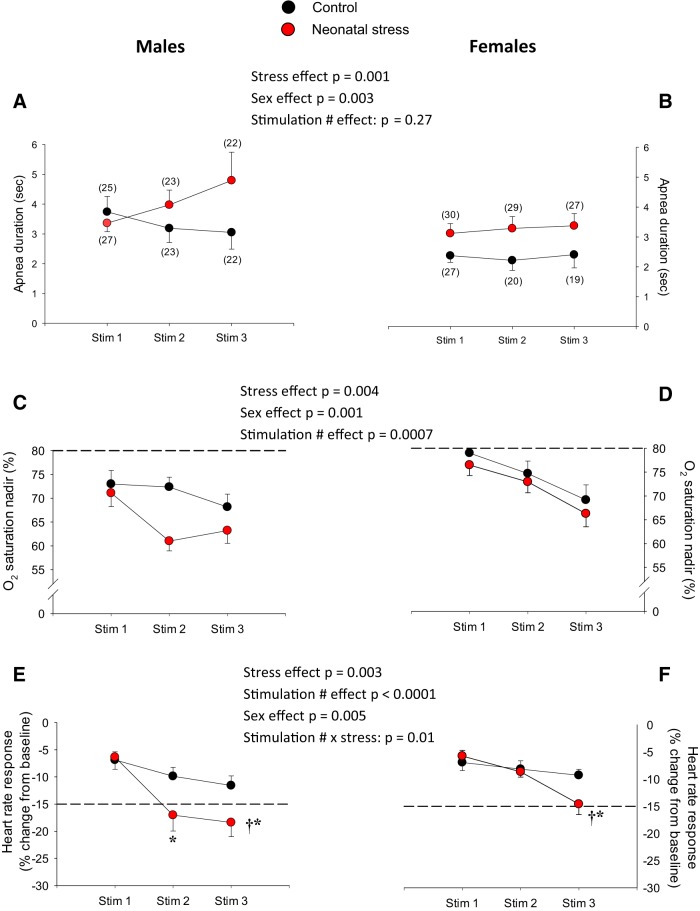
Neonatal stress augments cardio-respiratory inhibition following LCR stimulation. Top panels, Apnea duration for (***A***) males and (***B***) females. Middle panels, Nadirs in S*p* o_2_ observed in (***C***) males and (***D***) females. Bottom panels, Peak heart rate responses (bradycardias) observed in (***E***) males and (***F***) females. ***A***, ***B***, The numbers in brackets indicate the number of pups in which measurements were obtained; this applies for all cardio-respiratory variables. Experiments were performed at postnatal days 14 and 15. In the lower panels, the dotted lines indicate the S*p* o_2_ nadir (***C***, ***D***) and change in heart rate (***E***, ***F***) that are considered clinically significant in newborn (see Discussion for references and details). Black circles, pups that were not disturbed during the neonatal period (control); red circles, pups subjected to neonatal stress in the form of maternal separation (3 h/d, from postnatal days 3 to 12). Data are reported as mean ± SEM. For each variable, ANOVA results are reported in the figure; symbols indicate results from *post hoc* tests; *, statistically different from the value measured following the first stimulation (stim 1) at *p* < 0.05; †, statistically different from corresponding control value at *p* < 0.05.

Once respiratory activity was stable for at least 10 min, three water injections (10 µl each) were made using a 10-µl Hamilton syringe, starting at the beginning of inspiration. A 5-min recovery period was allowed between injections. Apnea duration was defined as the period of apneas/respiratory disruption from the beginning of the stimulus (water injection) until the return of at least five regular, uninterrupted breaths (Xia et al., 2008). S*p* o_2_ and heart rate were measured during baseline Figure 1 and 2. Based on animal studies using similar approaches (Cummings et al., 2009; Fournier et al., 2013), the O_2_ desaturation and bradycardia responses consisted of the lowest values achieved following water injection. Results were considered only when pups recovered before the next injection.

The fact that anesthesia impairs the mechanisms initiating breathing during LCR stimulation in piglets (Donnelly and Haddad, 1986) raises the possibility that the effect of anesthesia differs between treatments and sexes. However, evidence gathered to date indicate that the persistent and sex-specific effects of NMS on cardio-respiratory control are robust and that the effects observed in intact animals are similar to those obtained under anesthesia. Specifically, previous work performed on intact, unanesthetized pups show that NMS promotes respiratory instability and apneas and that this effect is more important in males (Gulemetova and Kinkead, 2011). Furthermore, experiments performed in adult male rats show that NMS-related enhancement of the O_2_ chemoreflex measured under urethane anesthesia is similar to the one observed in intact animals (Genest et al., 2004, 2007; Kinkead et al., 2005). Thus, the possibility that the phenotype reported here reflects an anesthesia related artifact is unlikely.

### Analysis of arterial blood-gases

Arterial blood gases influence LCR responsiveness (Xia et al., 2013). In a second series of experiments, pups were anesthetized as described previously and maintained at 35°C. A catheter pretreated with heparin was inserted in abdominal artery and a 70-µl blood sample was taken for analysis of arterial blood-gases (model ABL-5, Radiometer) corrected for the rat’s body temperature.

### Electrophysiological recordings

**Figure 3.**
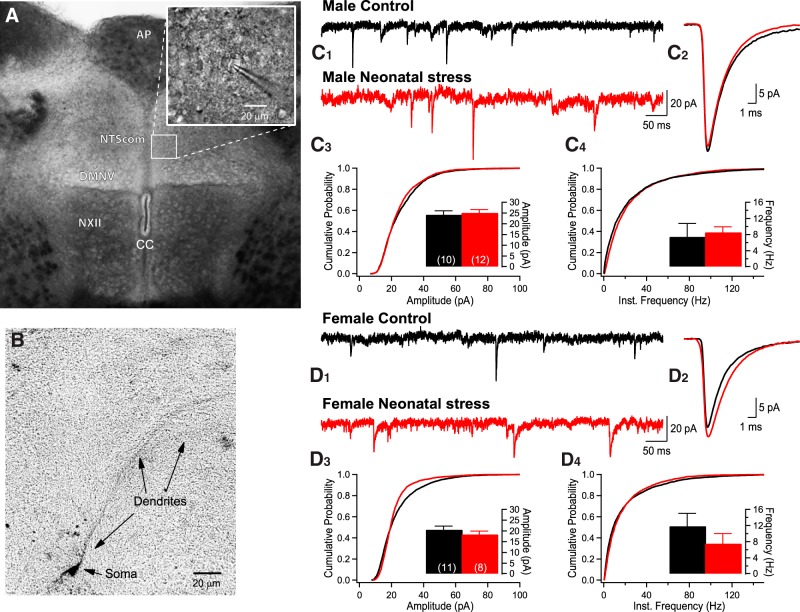
Neonatal stress has no effect on sEPSCs recorded in the NTScom. ***A***, Collage of photomicrographs illustrating the caudal brainstem slice in which whole-cell recordings were performed in the NTScom and the DMNV. The area postrema (AP), central canal (CC), and hypoglossal motor nucleus (NXII) NXII were used as visual landmarks. The inset shows a patch pipette attached to a cell. ***B***, Photomicrograph of a biocytin-labeled NTScom neuron in which recordings were obtained. ***C***, Comparison of EPSCs recorded in the NTScom of 14-d-old male pups that were either raised under standard conditions (black; control) or subjected to the neonatal stress protocol (red; NMS 3 h/d, postnatal days 3-12.). ***C_1_***, Original recordings. ***C_2_***, Superimposed average EPSCs (10-min recording) comparing group data. ***C_3_***, Cumulative probability plots of EPSC amplitudes; in the inset, the histograms show mean data from each cell ± SEM. Within each bar, the number in bracket indicates the number of cells that were recorded in this group. ***C_4_***, Cumulative probability plots of EPSC frequencies; in the inset, the histograms show mean frequency data from each cell ± SEM. ***D***, Comparison of EPSCs recorded in the NTScom of 14-d-old female pups (control vs neonatal stress). ***D_1_***, Original recordings. ***D_2_***, Superimposed average EPSCs (10-min recording) comparing group data. ***D_3_***, Cumulative probability plots of EPSC amplitudes with histograms presenting the mean data in the inset ± SEM. ***D_4_***, Cumulative probability plots of EPSC frequencies with histograms presenting the mean data in the inset ± SEM.

In a third series of experiments, tissue slices of the caudal medulla containing the commissural region of the nucleus of the solitary tract (NTScom) and the DMNV (Fig. 3*A*) were prepared for patch clamp recording.

### Rationale for approach and selection of medullary areas for recording

Retrograde labeling was not performed to avoid surgical stress as a confounding factor. Thus, we prioritized regions that (1) are relevant to LCR-related responses, (2) have homogenous cell morphology, and (3) can be easily and reliably identified with clear anatomic landmarks (Fig. 3*A*). The section level corresponds to Bregma -10.4 mm in the developing rat brain atlas (P14; Khazipov et al., 2015); the area postrema is a valuable landmark to reliably identify this specific area. Recordings were first performed in the NTScom which is one of the main synaptic relays of myelinated laryngeal afferents (Jordan, 2001). The slightly darker appearance region and the fact that it is located in the midline above the central canal facilitates identification and thus ensures repeatability of recording sites between experiments (Fig. 3*A*). The parasympathetic activity regulating heart rate arises from cardiac vagal neurons located in the nucleus ambiguus (NA) and DMNV (Mendelowitz, 1999; Dyavanapalli et al., 2014; Machhada et al., 2015). Although a greater proportion of parasympathetic neurons reaching the heart originate from the NA than the DMNV (Nosaka et al., 1979; Corbett et al., 1999), we chose to record from the DMNV because in addition to being adjacent to the NTScom, its lighter appearance by comparison with the hypoglossal motor nucleus (NXII) facilitates its identification (Fig. 3*A*). Moreover, the morphology and phenotype of neurons of the DMNV are relatively homogenous (Fox and Powley, 1992). Finally, abnormalities including delayed neuronal maturation, have been reported in the DMNV of SIDS victims (Quattrochi et al., 1985; O'Kusky and Norman, 1994; Bejjani et al., 2013).

### Medullary slice preparation

Slices from P14–P15 rats were prepared according to established procedures (Evstratova et al., 2014). Briefly, pups were anesthetized with isoflurane and decapitated. The brain was promptly removed from the skull and immersed in ice-cold artificial CSF (aCSF) previously equilibrated with 95% O_2_-5% CO_2_ containing 87 mM NaCl, 25 mM NaHCO_3_, 2.5 mM KCl, 1.25 mM NaH_2_PO_4_, 7 mM MgCl_2_, 0.5 mM CaCl_2_, 25 mM glucose, and 75 mM sucrose. The medulla was dissected and frontal slices (300 µm) were cut with a vibratome (Leica Model VT1000S). Slices were then placed in oxygenated aCSF at 32°C for at least 30 min and then kept at room temperature until they were used for experiments. All recordings were performed using aCSF containing 124 mM NaCl, 25 mM NaHCO_3_, 2.5 mM KCl, 1.5 mM MgCl_2_, 2.5 mM CaCl_2_, and 10 mM glucose (95% O_2_-5% CO_2_; pH 7.4).

### Electrophysiological recordings

#### Series I. Standard conditions

Medullary slices containing the regions of interest were transferred to a recording chamber and secured with a nylon mesh and superfused with aCSF at a flow rate of 2 ml/min and maintained at 32-34°C (TC-324C Heater Controller; Warner Instruments). Whole-cell voltage-clamp recordings were made from neurons of the NTScom and DMNV (Fig. 3*A*). Neurons were visualized under an upright Olympus microscope using a 40× water-immersion objective, equipped with differential interface contrast and an infrared-sensitive camera (Olympus XM 10). Glass electrodes (4–6.5 MΩ) were filled with a solution containing 120 mM K-gluconate, 20 mM KCl, 2 mM MgCl_2_, 0.6 mM EGTA, 2 mM Mg_2_ATP, 0.3 mM NaGTP, 10 mM HEPES, and 7 mM phosphocreatine (285-290 mOsm) and supplemented with biocytin in a subset of experiments.

Spontaneous EPSCs (sEPSCs) were recorded using an Axopatch 200B amplifier (Molecular Devices) operating under voltage-clamp mode. Data were filtered at 2 kHz and acquired using pCLAMP 10 software (Molecular Devices). Recordings were performed at a holding potential of -60 mV. The uncompensated series resistance was monitored by the delivery of 5-mV steps throughout the experiment, only recordings with <20% change over the course of the protocol were analyzed. We measured and monitored the access resistance in all recordings. Neurons recorded in the NTScom had an average access resistance of 23.7 ± 1 MΩ (*n* = 41 neurons). Neurons recorded in the DMNV had an average access resistance of 12.38 ± 0.49 MΩ (*n* = 54 neurons). The different access resistance is consistent with the fact that we typically used smaller pipettes for NTScom neurons, as they are smaller. It was easier to target the NTScom neurons with smaller electrodes than with larger electrodes, hence the large range in the pipette size that we used (4–6.5 MΩ).

Recording was performed under basal conditions for 10 min. sEPSCs represent both action potential-driven synaptic currents and miniature synaptic currents, which reflect the spontaneous release of glutamate from individual synaptic sites. Thus, aCSF containing tetrodotoxin (TTX; 1.25 µM) was then applied onto the slice to abolish presynaptic activity and record action potential-independent mEPSCs. In the present context, comparison of EPSCs before and after TTX application allows to functionally distinguish between changes in the number of synaptic inputs versus changes in synaptic strength. The TTX-containing aCSF was washed over a 10-min period, following which mEPSCs were recorded over a 10-min period.

#### Series II. Testosterone exposure

Given the sex-specific effects of stress on physiologic responses, EPSCs in the DMNV, and the positive correlation between testosterone and corticosterone in stressed male animals, we next evaluated androgen receptor signaling as a potential mechanism to modulate excitatory synaptic signaling in the DMNV. Notably, androgen receptors are expressed in the DMNV (Mukudai et al., 2016) and androgen receptor signaling was previously shown to modulate synaptic transmission in the hippocampus (MacLusky et al., 2006). In these experiments, medullary slices recovered from the sectioning procedure in the presence o*f* testosterone [10 nM in dimethyl sulfoxide (DMSO) 0.004%; Sigma-Aldrich] or vehicle (DMSO) for 1 h before recording. This concentration is based on peak levels measured in developing male rats (Corbier et al., 1978; Weisz and Ward, 1980) and concentrations used in *in vitro* studies (Ito et al., 1999).

### Corticosterone and testosterone measurements

Following deep anesthesia with ketamine/xylazine (intraperitoneal), terminal blood samples by intracardiac puncture (∼2.5 ml) were obtained from pups according to standard laboratory procedures (Fournier et al., 2013). Note that due to technical problems, not all samples were analyzed for both hormones. Samples for corticosterone analysis were placed in a microvette 500K3 EDTA tube and samples for testosterone analysis were placed in one serum-gel clotting activator Microtub (Starsted). Serum-gel tubes were kept at room temperature for 30 min before centrifugation with EDTA tubes (13,000 rpm, 4°C for 10 min). After centrifugation, serum and plasma were collected and place in a −80°C freezer until assayed.

Analysis of corticosterone and testosterone were performed in duplicates as we have done previously, (Genest et al., 2004; Fournier et al., 2013) using immunoassay (ELISA) kits (Enzo Life Sciences) and a microplate spectrophotometer (µQuant, Bio-Tek Instruments). Concentrations were calculated from the parameters of the standard curve linearized by a log-log transformation.

### Experimental design and statistical analysis

Data obtained from stressed animals are compared to that of controls. Each group is composed of multiple litters to avoid litter specific effects. As a result, the number of replicates used here (>19 pups/group) is substantial and exceeds what is commonly used in the vast majority of physiologic studies but ensures robust statistical analyses. Males and females were used in all experiments.

The results were analyzed using a multifactorial ANOVA to assess effect of neonatal stress (NMS), sex; the effect of repeated injections was considered for LCR analysis. In some electrophysiological experiments data were compared between the NTScom and the DMNV (structure effect). Note that statistical analyses of S*p* o_2_ nadirs and bradycardias were performed without baseline data. Because some pups did not survive the entire protocol, a non-repeated measures design was used. The numbers that appear in bracket over the symbols in Figure 2*A*,*B* give the number of replicates in each group and thus provides detailed information about the number of deaths and repartition among groups. Statistical analysis revealed no significant effect of stress or sex on mortality rate. When ANOVA results indicated that a factor (or interaction between factors) was significant (*p* < 0.05), a Fisher’s least significant difference test was performed for *post hoc* analysis.

Automated analyses of sEPSC frequency and amplitude were performed on 250 events per cell using the “ClampFit” module of the recording software. The detection threshold was set at three times the SD of the baseline noise (Clements and Bekkers, 1997) so that no EPSCs were under 6.2 pA were considered in the analysis. This approach avoids the detection of false positives. Cumulative probability plots are reported to illustrate data distribution; however, the results were averaged to produce a mean value for each animal so that a multifactorial ANOVA on mean group data could be performed also. This approach was favored because it is more conservative and makes it possible to assess the effects of stress, sex, drug application (TTX, testosterone), and factorial interactions on EPSC frequency and amplitude.

Correlations between testosterone and corticosterone were first analyzed using the least square method and *z* test. The effects of stress, sex, and corticosterone levels on testosterone levels were then evaluated with an analysis of covariance (ANCOVA). Statistical analyses were performed using StatView 5.0 (SAS Institute). Data are reported as mean ± SEM. ANOVA results are mainly reported in the figures and Table 1. Results from *post hoc* tests are represented by symbols in the table and figures.

**Table 1.** Comparison of body weight, selected cardio-respiratory variables, and arterial blood gases between pups maintained under standard conditions (control) versus those subjected to neonatal stress (maternal separation)

	Control		Neonatal stress				
	Female	Male	Female	Male	Stress effect	Sex effect	Factorial interaction
Body weight (g)	41 ± 1.4	41 ± 1.3	37 ± 1.2	37 ± 1.4	***p* = 0.003** *F*_(1,124)_ = 9.408	*p* = 0.49 *F*_(1,124)_ = 0.469	*p* = 0.96 *F*_(1,124)_ = 0.002
Respiratory frequency (breaths/min)	51 ± 2.9	51 ± 3.5	47 ± 2.4	47 ± 2.7	*p* = 0.17 *F*_(1,120)_ = 1.895	*p* = 0.98 *F*_(1,120)_ = 0.001	*p* = 0.98 *F*_(1,120)_ = 0.001
SpO_2_ (%)	91 ± 1.0	90 ± 1.2	90 ± 1.1	88 ± 1.2	*p* = 0.11 *F*_(1,119)_ = 2.601	*p* = 0.19 *F*_(1,119)_ = 1.745	*p* = 0.69 *F*_(1,119)_ = 0.160
Heart rate (beats/min)	414 ± 15	418 ± 15	394 ± 12	430 ± 16	*p* = 0.79 *F*_(1,120)_ = 0.066	*p* = 0.29 *F*_(1,120)_ = 1.151	*p* = 0.17 *F*_(1,120)_ = 1.892
Body temperature (^o^C)	35.0 ± 0.0	35 ± 0.0	34.9 ± 0.0	35.0 ± 0.0	*p* = 0.34 *F*_(1,116)_ = 0.935	*p* = 0.34 *F*_(1,116)_ = 0.935	*p* = 0.34 *F*_(1,116)_ = 0.935
pHa	7.25 ± 0.03	7.27 ± 0.03	7.33 ± 0.03	7.36 ± 0.03	***p* = 0.01** *F*_(1,24)_ = 7.072	*p* = 0.51 *F*_(1,24)_ = 0.456	*p* = 0.85 *F*_(1,24)_ = 0.035
PaO_2_ (mm Hg)	97 ± 5.9	99 ± 5.6	102 ± 8.9	100 ± 4.4	*p* = 0.62 *F*_(1,25)_ = 0.250	*p* = 0.94 *F*_(1,25)_ = 0.006	*p* = 0.78 *F*_(1,25)_ = 0.082
PaCO_2_ (mm Hg)	38 ± 3.5	40 ± 4.1	32 ± 2.2	30 ± 3.1	***p* = 0.01** *F*_(1,25)_ = 7.351	*p* = 0.98 *F*_(1,25)_ = 0.0005	*p* = 0.57 *F*_(1,25)_ = 0.334

Measurements were performed in anesthetized pups (14-15 d old) under resting conditions. Note that blood gas measurements were obtained in a distinct series of experiments; LCR in these pups was not tested. Data are reported as mean ± SEM. A bold value indicates a significant effect of factors (ANOVA).

## Results

Body weight of pups subjected to stress were, on average, 9.8% lighter than controls; sex-based differences were not observed. Before LCR stimulation, cardio-respiratory variables and body temperature were similar between groups (Table 1). By comparison with controls, stressed pups were hypocapnic and alkalotic. Arterial blood gases did not differ between males and females.

### Neonatal stress increases severity of cardio-respiratory responses to LCR stimulation

Water injection near the larynx activated the LCR (Fig. 1) and provoked both cardio and respiratory inhibition. Thus, the drop in S*p* o_2_ results not only from the apnea but also the cardio-vascular responses resulting from para-sympathetic activation. The cardio-respiratory inhibition observed in NMS pups was generally greater than controls. The recording reported in Figure 1 was obtained following the third stimulation when group differences were more important. Comparison of population data show that in stressed pups, LCR-related apneas lasted longer than controls (Fig. 2*A*,*B*). Overall, the duration of apneas was longer in males but was not influenced by multiple stimulations. The S*p* o_2_ nadirs reached following LCR stimulation were lower in stressed pups; the lowest S*p* o_2_ nadirs were observed in stressed males (Fig. 2*C*,*D*). Assessing the specific effects of repetitive water injection showed that the S*p* o_2_ minima reached during apneas decreased over the course of the protocol. Heart rate decreased following LCR stimulation; expressing heart rate responses as a percentage change from baseline showed that the largest bradycardias were measured in stressed pups, especially in males (Fig. 2*E*,*F*). Unlike controls, the intensity of the bradycardias measured in stressed pups increased over the course of the protocol. Note that in Figure 2, the dotted lines that appear in panels *C–F* indicate the level of S*p* o_2_ nadirs and bradycardia considered clinically significant (Eichenwald et al., 2016).

### Heterogeneous effects of sex and stress on EPSCs in pups

In Figure 3, panels *C_1_* and *D_1_* compare sEPSCs recordings from NTScom cells in each experimental group. In panels *C_3_* and *C_4_* and *D_3_* and *D_4_*, the cumulative probability plots for interevent intervals illustrate data distribution, whereas the histograms represent mean data. Superimposed average EPSCs for each group are compared in panels *C_2_* and *D_2_*. Stress had no significant effect on these EPSCs (amplitude: *F*_(1,37)_ = 0.150; *p* = 0.70; frequency: *F*_(1,37)_ = 0.404; *p* = 0.53). However, the amplitude of the sEPSCs recorded in the NTScom were, on average, 20% greater in males than females (sex effect: *F*_(1,37)_ = 9.226; *p* = 0.004; Fig. 5*C_2_*).

**Figure 4.**
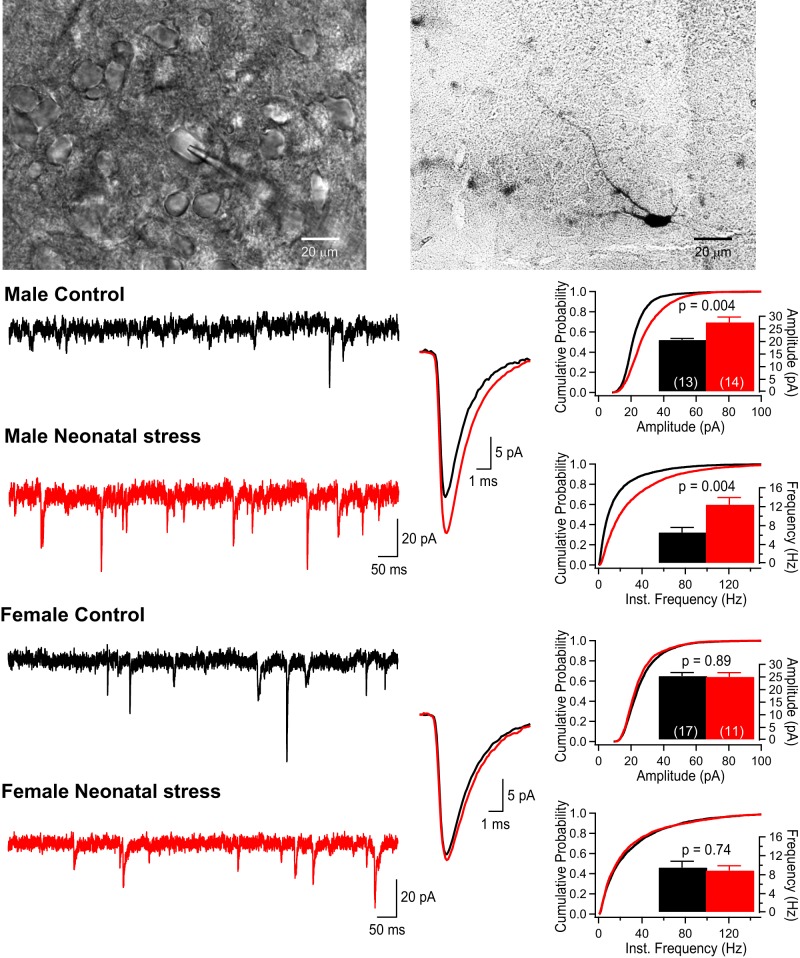
Neonatal stress induces sex-specific augmentation of sEPSCs in the DMNV. ***A***, Photomicrograph of a patch pipette attached to a cell within the DMNV. ***B***, Photomicrograph of a biocytin-labeled DMNV neuron in which recordings were obtained. ***C***, Comparison of EPSCs recorded in the DMNV of 14-d-old male pups that were either raised under standard conditions (black; control) or subjected to the neonatal stress protocol (red; NMS 3 h/d, postnatal days 3–12). ***C_1_***, Original recordings. ***C_2_***, Superimposed average EPSCs (10-min recording) comparing group data. ***C_3_***, Cumulative probability plots of EPSC amplitudes; in the inset, the histograms show mean data ± SEM for each group. Within each bar, the number in bracket indicates the number of cells that were recorded in this group. ***C_4_***, Cumulative probability plots of EPSC frequencies; in the inset, the histograms show mean frequency data ± SEM for each group. ***D***, Comparison of EPSCs recorded in the DMNV of 14-d-old female pups (controls vs neonatal stress). ***D_1_***, Original recordings. ***D_2_***, Superimposed average EPSCs (10-min recording) comparing group data. ***D_3_***, Cumulative probability plots of EPSC amplitudes with histograms presenting the mean data ± SEM in the inset. ***D_4_***, Cumulative probability plots of EPSC frequencies; *p* values reported above the histograms are results from *post hoc* tests.

Figure 4*C_1_*,*D1* compares sEPSC recordings obtained in the DMNV. Superimposed average EPSCs for each group are compared in panels Figure 4*C_2_*,*D_2_*. ANOVA shows that neonatal stress augmented the amplitude (Fig. 4*C_2_*,*C_3_*) and frequency (Fig. 4*C_4_*) of EPSCs in males but not females (Fig. 4*D*); the factorial interaction between stress and sex was significant for both variables (amplitude: *F*_(1,52)_ = 5.608; *p* = 0.02; frequency: *F*_(1,52)_ = 6.195; *p* = 0.02).

DMNV neurons show autonomous pacemaker properties owing to the presence of a voltage- and TTX-insensitive leak sodium current (Goldberg et al., 2012). Consequently, the SD of the baseline signal (noise level) of DMNV recordings (2.07 ± 0.04 pA, *n* = 54) is slightly higher than in the NTScom (1.62 ± 0.06, *n* = 40, structure effect: *F*_(1,92)_ = 38.521; *p* < 0.0001). ANOVA showed that the noise level was not stress or sex dependent.

### Neonatal stress augments EPSC amplitude response to TTX application in the DMNV

**Figure 5.**
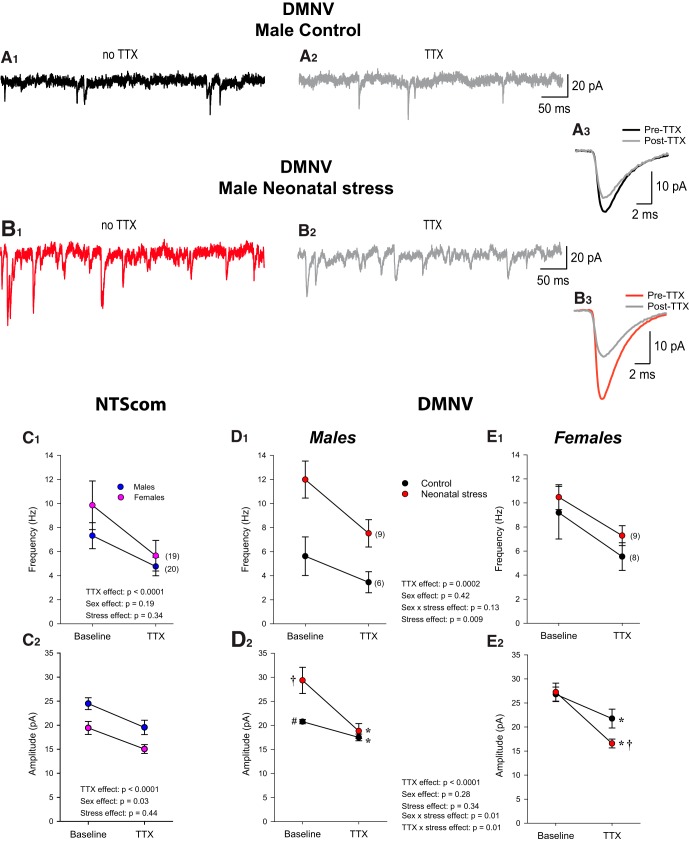
Heterogeneous effects of neonatal stress on the EPSC responses to TTX application. Representative EPSC recording obtained within the DMNV of male control pups (***A_1_***) before (baseline; black) and (***A_2_***) after TTX application (gray); recording is from the same neuron. ***A_3_***, Superimposed average EPSCs (10-min recording) comparing data before and after TTX application. Similar recording from a neuron from a male subjected to neonatal stress (***B_1_***) before (red) and (***B_2_***) after TTX application (gray). ***B_3_***, Superimposed average EPSCs (10-min recording) for each condition. Mean EPSC data from the NTScom for (***C_1_***) frequency (***C_2_***) and amplitude before and after TTX application. For each variable, ANOVA results for each factor (and factorial interactions) appear in the graphs. In the NTScom, stress had no effect on either variable; data from control and stressed groups were pooled. Because the effect of sex was significant for amplitude, data from males (blue circles) and females (red circles) are reported separately. Within the DMNV, mean EPSC frequency (***D_1_***, ***E_1_***) and amplitude data (***D_2_***, ***E_2_***) are reported separately for each group and sex since the responses to TTX application were influenced by both factors. Data are reported as mean ± SEM; the numbers in brackets indicate the number of cells in each group. Symbols indicate results from *post hoc* tests; *, statistically different from the baseline (pre-TTX) value at *p* < 0.05; †, statistically different from corresponding control value at *p* < 0.05; #, statistically different from corresponding female value at *p* < 0.05.

Comparison of EPSC recordings before and after TTX application show that in the DMNV, the effects of TTX were generally greater in cells from stressed pups than controls (Fig. 5*A*,*B*,*D*). This effect of stress was not observed in the NTScom (Fig. 5*C_1_*,*C_2_*). The amplitude of the EPSCs recorded in the NTScom before TTX application were not influenced by stress but were greater in males than females (Fig. 5*C_2_*). TTX application reduced both the frequency and amplitude of EPSCs; however, the magnitude of this effect was not influenced by stress or sex (Fig. 5*C_1_*,*C_2_*). In the DMNV, TTX application also decreased the frequency and amplitude of EPSCs (Fig. 5*A*,*B*). While the effect of TTX on EPSC frequency did not differ between groups (Fig. 5*D_1_*,*E_1_*), the reduction in EPSC amplitude was larger in stressed pups (both males and females; Fig. 5*D_2_*,*E_2_*, respectively). Note that in males, EPSC amplitude before TTX application was higher in stressed cells than control and that TTX brought these values to a similar level (Fig. 5*D_2_*). Conversely, EPSC amplitudes of females were similar under baseline conditions but TTX application then revealed significant group differences (Fig. 5*E_2_*).

### Neonatal stress influences the relationship between corticosterone and testosterone levels in pups

**Figure 6.**
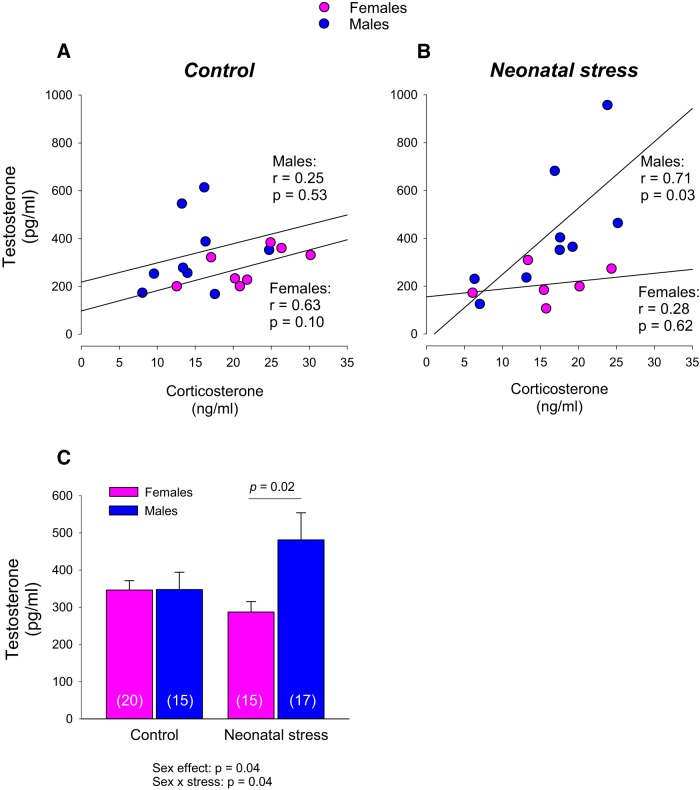
Neonatal stress has sex-specific effects on the relationship between circulating testosterone and corticosterone levels in pups. Comparison of the correlations between corticosterone and testosterone in pups maintained in (***A***) control conditions or (***B***) subjected to neonatal stress in the form of maternal separation (3 h/d, from postnatal days 3 to 12). Data are reported for males (blue) and females (pink). ***C***, Comparison of mean testosterone levels between control and stressed pups. Data are reported as mean ± SEM. Numbers in brackets within the bars indicate the number of samples analyzed in each group. ANOVA results are reported below the histogram. Note that the number of samples used for this panel is greater than in ***A***, ***B*** because corticosterone values could not be obtained for some samples.

NMS augments circulating corticosterone levels; this effect persists at least 24 h after the last separation procedure (Gulemetova and Kinkead, 2011). Here, measurements performed at P14–P15, show that corticosterone levels of NMS pups were no longer higher than controls (stress effect: *F*_(1,31)_ = 0.601; *p* = 0.44; for individual data, see Fig. 6*A*,*B*). Testosterone levels measured in males were higher than females; this difference was most noticeable in stressed pups (Fig. 6*C*). In control pups, there was no relationship between the corticosterone and testosterone, regardless of sex. In stressed pups, however, testosterone levels correlated positively with corticosterone; this relationship was observed in males only. ANCOVA confirmed that corticosterone had a significant influence on testosterone (corticosterone: *F*_(1,24)_ = 6.105; *p* = 0.021).

### Sex-specific effects of testosterone on EPSCs in the DMNV

**Figure 7.**
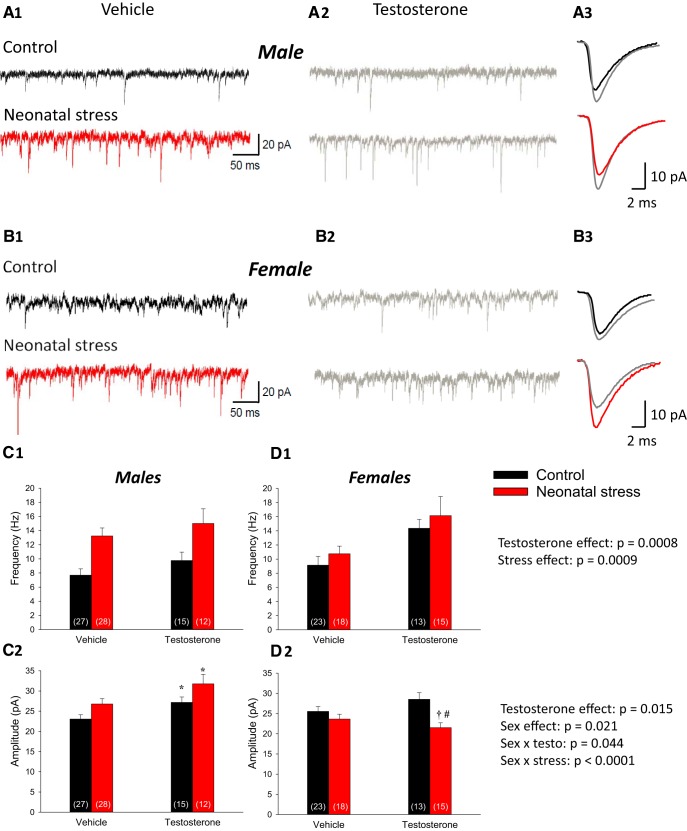
Sex-specific effects of testosterone incubation on the EPSC in the DMNV. Comparison of EPSC recordings obtained within the DMNV from tissue slices were either incubated in vehicle (***A_1_***; DMSO 0.004%, colored) or testosterone (***A_2_***; 10 nM, gray) for 1 h before recordings. Data were obtained in male pups raised in control conditions (black) or subjected to neonatal stress (maternal separation; red). ***A_3_***, Superimposed average EPSCs (10-min recording) comparing data with and without testosterone incubation. Similar recordings from neurons from females in which tissue was previously incubated with vehicle (***B_1_***) or testosterone (***B_2_***). ***B_3_***, Superimposed average EPSCs (10-min recording) for each condition. Mean EPSC data from males comparing (***C_1_***) frequency (***C_2_***) and amplitude with or without testosterone preincubation application in pups raised in control conditions or subjected to neonatal stress. Similar data are reported for females in ***D_1_***, ***D_2_***. ANOVA results for each factor (and factorial interactions) appear in the graphs. Data are reported as mean ± SEM; the numbers in brackets indicate the number of cells in each group; *, statistically different from the vehicle value at *p* < 0.05; **†,** statistically different from corresponding control value at *p* < 0.05; #, statistically different from corresponding male value at *p* < 0.05.

We first compared the amplitude and frequency of EPSCs reported previously (standard condition) with those incubated with vehicle. Since results showed no effect of DMSO (amplitude: *F*_(1,89)_ = 1.244; *p* = 0.27; frequency: *F*_(1,89)_ = 1.767; *p* = 0.19; not shown), data were pooled to increase statistical power. We then observed that testosterone incubation significantly increased the frequency of EPSCs (testosterone effect: *F*_(1143)_ = 11.844; *p* = 0.0008; Fig. 7*A_1_*,*A_2_*,*B_1_*,*B_2_*,*C_1_*,*D_1_*); this effect was not influenced by stress or sex (Fig. 7). Next, the EPSC amplitude was also significantly increased by testosterone incubation (testosterone effect: *F*_(1143)_ = 6.008; *p* = 0.015). However, this effect was sex-specific, that is significant in males, but not females (testosterone × sex: *F*_(1143)_ = 4.107; *p* = 0.044; Fig. 7*A3*,*B3*,*C2*,*D2*). We noted that testosterone incubation increased the EPSC amplitude in both control and stressed males by a similar factor (18% and 19%, respectively). Interestingly, testosterone incubation of slices from control animals increased the EPSC amplitude to a level similar to non-incubated slices from stressed animals. In contrast, testosterone incubation of either control or stressed female rats had no significant effect on the EPSC amplitude, hereby suggesting that androgen receptor activity has sex-specific consequences in the DMNV (Fig. 7).

## Discussion

In immature mammals, the presence of liquid near the larynx stops breathing to prevent aspiration of foreign substances into the lungs. In humans, prolonged apneas, profound O_2_ desaturations, and bradycardias resulting from an immature/abnormal LCR can ultimately be fatal (Kinney and Thach, 2009). Here, we show that subjecting pups to an apparently mild stressor (maternal separation) during a critical period of development is sufficient to augment cardio-respiratory inhibition following LCR activation, especially in males. Comparisons with humans requires caution but the majority of desaturations and many bradycardias observed following LCR stimulation would be considered clinically significant, especially in stressed males (Eichenwald et al., 2016). Comparisons of arterial blood gases show that stressed pups are alkalotic and EPSC recordings indicate that synaptic inputs converging on the DMNV are increased in stressed males. Together, these data provide plausible explanations for the longer apneas, larger bradycardias, and O_2_ desaturations observed in stressed pups.

Several hormones can influence respiratory control during early life; however, we opted to focus on testosterone because perinatal stress disrupts androgen secretion in newborn (Weisz and Ward, 1980; Fournier et al., 2013). This effect is significant as testosterone contributes to brain masculinization by inducing or repressing synapse formation in a region-specific manner during early life (Schwarz and McCarthy, 2008; Lenz et al., 2012). The demonstration that in stressed males, circulating testosterone is proportional to corticosterone levels reveals a significant neuroendocrine disruption. These results point to new and plausible hypotheses regarding the origins of sex-specific cardio-respiratory dysfunction during development.

### Neonatal stress augments LCR-induced cardio-respiratory inhibition

Variations in respiratory drive influence the LCR, especially apnea duration (Xia et al., 2013). Since experimental conditions were strictly controlled, results showing that stressed pups were alkalotic and hypocapnic were unexpected. This acid-base disturbance likely contributes to the prolonged apneas but because the basal cardio-respiratory variables reported here or in nonanesthetized pups (Gulemetova and Kinkead, 2011) do not differ between groups, the overall impact on respiratory drive is not proportional to the magnitude of alkalosis/hypocapnia (Table 1). The origin of this hypocapnia is unknown. However, because neurons of the NTS and DMNV also control various gastroesophageal reflexes that remove CO_2_ from blood (Dean, 2011), results from electrophysiological experiments suggest that stress could have promoted CO_2_ excretion via this alternate route. Alternately, insufficient metabolism of enteric urea has been evoked to explain the alkalosis observed in SIDS victims (Wiklund et al., 1998).

Because multiple water applications do not influence LCR intensity (Xia et al., 2008), repeated stimulations were performed with the intention to obtain a more representative “mean response” for each animal. Although valid in controls, this approach would have been misleading in stressed males in which O_2_ desaturations and bradycardias augmented over the course of the protocol. These effects suggest that neonatal stress compromises recovery. In this population, the risk of cardio-respiratory failure may increase with repeated LCR activation.

### Regional heterogeneity and sex-specific impact of neonatal stress on EPSCs

The NTScom is an important synaptic relay for various sensory inputs, including those from the laryngeal mucosa (Jordan, 2001; Wang et al., 2016). In rats, laryngeal afferents activate secondary NTS neurons that then bifurcate to cells regulating somatic functions, including control of airway closure, inspiratory inhibition, and preganglionic para-sympathetic cells that induce bradycardia (Nosaka et al., 1979; Furusawa et al., 1996; Mendelowitz, 2000; Praud, 2010; Wang et al., 2016). In the NTScom, quantification of EPSCs before and after TTX application did not differ between groups, thus indicating that stress did not alter excitatory synaptic inputs converging onto this region. It is therefore unlikely that differences in sensory afferents converging onto the NTScom are responsible for stress-induced augmentation of the LCR. However, the larger sEPSC amplitudes observed in males without concomitant change in basal frequency or sensitivity to TTX application reveal sex-based differences in postsynaptic mechanisms in this structure (Cormier and Kelly, 1996). The NTScom data are consistent with our demonstration that LCR intensity is, overall, greater in males than females regardless of stress exposure. Since testosterone augments AMPA receptor expression in the hypothalamus (Diano et al., 1997), this postsynaptic effect may also occur within medullary regions regulating the LCR.

Within the DMNV, neonatal stress augmented sEPSC frequency and amplitude in males but not females (Fig. 4). This is a significant observation since, to the best of our knowledge, it is the first demonstration of a sex-specific effect of stress within the CNS at the cellular level. Furthermore, these data provide a reasonable explanation for the sex-specific enhancement of bradycardias and O_2_ desaturations observed in stressed pups. To gain insight into the synaptic upregulation observed in males, we used TTX application to analyze sEPSCs (pre-TTX) and quantal (post-TTX) EPSCs for control and stressed pups. In males, we observed equal quantal size between groups, thereby indicating a change in the number of release sites (active synapses) or the release probability to support larger sEPSCs. Consistent with this idea, we observed a larger frequency of sEPSCs in stressed compared to control animals (Fig. 5*D1*). Differences in mEPSC frequency can distinguish between increases in release probability versus the number of active synapses. Here, TTX application significantly and similarly decreased the frequency of mEPSC in both control and stressed pups. The mEPSC frequency observed in stressed pups remained larger than in controls, thereby suggesting a larger number of active release sites. This effect of stress on frequency was not observed in females. Instead, analysis of mEPSCs (post-TTX) revealed a smaller quantal size for stressed females than for controls, an observation that further highlights sex-specific effect of neonatal stress on neuronal networks. Accordingly, we propose that females are more resilient to the effects of stress, potentially because of initially different configurations and/or ovarian hormones.

Within the DMNV, stress augments synaptic function both at the pre- and postsynaptic level. The differences between regions and sexes remain unexplained but could reflect the heterogeneity of corticosterone receptor distribution within the medulla (Morimoto et al., 1996) and/or sexual differentiation. This developmental process varies between brain regions and involves different mechanisms, including perinatal testosterone secretion (and its derivative estradiol; Lenz and McCarthy, 2010). Considering the argument developed previously, it is conceivable that testosterone augments AMPA receptors (and thus increases sEPSC amplitude) in the DMNV of stressed males. The increased propensity of stressed pups to secrete more testosterone during stress supports this interpretation and acute incubation of medullary slices with testosterone experiments aimed to test this basic concept. Admittedly, the effects of stress on testosterone secretion during development are more complex than the protocol used here. Yet, this simple paradigm showed that androgen receptor signaling in the DMNV is sufficient to increase excitatory synaptic activity, especially in males. We therefore propose that elevated testosterone contributes to the sex-specific effects of stress on excitatory synaptic activity we reported in this region.

Analysis of sEPSCs and mEPSCs provides functional evidence that synaptic inputs converging onto DMNV neurons are more numerous in stressed pups, and thus likely contribute to the larger LCR in these animals. This result is consistent with more numerous dendritic spines within that region and coincidentally, the mean synapse and spine density in the DMNV of SIDS victims are significantly greater than controls (Quattrochi et al., 1985). Of note, corticosterone is an important regulator of dendritic spine formation (Liston and Gan, 2011) and circulating levels are augmented following NMS; this response does not differ between sexes (Gulemetova and Kinkead, 2011).

### Sex-specific influence of neonatal stress on the relationship between testosterone and corticosterone

In the adult rat, there are extensive interactions between the hypothalamic-pituitary-adrenal (HPA) and the hypothalamic-pituitary-gonadal (HPG) axes. Acute stress increases testosterone secretion which in turn, attenuates HPA axis activity. However, chronic stress has the opposite effect (Foilb et al., 2011). This relationship undergoes substantial developmental changes such that before 40 d of age, stress exposure does not increase testosterone level, an effect attributed to a greater negative feedback by testosterone (Foilb et al., 2011). Hormone measurements from control pups and stressed females are in line with these reports, thus confirming the immaturity of neuroendocrine function at P14–P15. In stressed males, however, the positive relationship between corticosterone and testosterone indicates abnormal development. Consequently, the testosterone levels achieved during and/or following NMS in males were likely higher than in the other groups. Testosterone has a significant impact on brain development. Although correlative, these results are nonetheless significant as they provide a reasonable explanation for the sex-specific effects of stress on LCR.

### Perspectives

Perinatal exposures to adverse environmental conditions play a significant role in the etiology of SIDS (Kinney and Thach, 2009). Unlike clinical research, the rat allows better control over the newborn’s environment and eliminates confounding factors such as maternal lifestyle and socio-economic status. Here, we showed that an apparently modest stress (NMS) that poses no direct threat to respiratory homeostasis is sufficient to augment LCR-induced cardio-respiratory inhibition, especially in males. Interestingly, this result, combined with the male bias, abnormalities in the DMNV, alkalosis, and increased propensity to secrete testosterone have been reported in SIDS victims. Considering that established disruptors of respiratory development such as hypoxia, nicotine, or cocaine are stressors that can activate the neuroendocrine response to stress in rodents (Damianopoulos and Carey, 1995; Stanulis et al., 1997; Raff et al., 2003; Lutfy et al., 2012), our results bring us to propose that the release of stress hormones in developing newborn is a common mechanism in the etiology of SIDS. Together, these observations bring valuable support to the hypothesis that stress-related disruption of testosterone secretion during early life contributes to the increased prevalence in cardio-respiratory disorders in males, including SIDS (Rousseau et al., 2017).

## Synthesis

Reviewing Editor: Quentin Pittman, University of Calgary Decisions are customarily a result of the Reviewing Editor and the peer reviewers coming together and discussing their recommendations until a consensus is reached. When revisions are invited, a fact-based synthesis statement explaining their decision and outlining what is needed to prepare a revision will be listed below.

Your manuscript is improved as a result of revisions. However, the reviewers and I have carefully assessed both the revised version and your response to the reviewers and have one major item and a couple of minor issue that must be dealt with for the manuscript to be judged acceptable.

It is necessary for you to correct your statistical analysis as described in this comment: “In Table 1, and Figs 2, 5 and 7, the authors insert symbols to indicate significant differences between treatment groups even though there were no between factors interactions. In their rebuttal to Reviewer 1's comment (#1), they claim that despite no significant interactions, their understanding is that post hoc tests are justified for each factor, if at least two factors have significant effects. It is not appropriate to do post hoc tests when no significant interactions are found. This is misleading as it causes the reader to draw erroneous conclusions. For example, in Table 1, the authors found a significant effect of stress on pHa, but no effect of sex and further no interaction between sex and stress, and so the only conclusion that can be made is that neonatal pups in general have higher levels of pHa than control animals, independent of sex. The presence of a symbol, however, now causes one to draw the erroneous conclusion that stressed males have significantly higher levels of pHa than control males but the difference between stressed and control females is not significantly, even though this difference is 0.8 compared to a difference of 0.9 between the males. The same is true in the Figures mentioned. Also, if the written description of the results is stated correctly, why use symbols that are misleading on the figures, that could lead to erroneous conclusions.” This must be corrected..

2. Your rebuttal to comment 2 of reviewer 2 ( interrupted breathing) could be included in the manuscript as other readers may miss this point.

3. Although additional experiments are not required, it is necessary for you to discuss the possibility that hormones other than testosterone could be involved and your conclusions should be amended to reflect that you have correlations and not necessarily causality

Minor suggestions for revision:

1. Page 1, line 25, change factor to factors.

2. Table 1, column 7, change sex effet to sex effect.

3. Figure 2A, consider changing Stimulation effect to Stimulation # effect to be consistent.

4. Figure 6A, it would be nice to use the same x-axis scale as 6B, it causes less confusion when comparing the two graphs

5. ) Line 456 ...This effect was not observed in females... The sentences previously regarding the males was referring to the frequency. Are the sentences regarding the females referring to the frequency or amplitude? Please clarify.

## Author Response

RESPONSE TO REVIEWERS

Synthesis Statement for Author (Required):

Your manuscript is improved as a result of revisions. However, the reviewers and I have carefully assessed both the revised version and your response to the reviewers and have one major item and a couple of minor issue that must be dealt with for the manuscript to be judged acceptable.

It is necessary for you to correct your statistical analysis as described in this comment: “ In Table 1, and Figs 2, 5 and 7, the authors insert symbols to indicate significant differences between treatment groups even though there were no between factors interactions. In their rebuttal to Reviewer 1's comment (#1), they claim that despite no significant interactions, their understanding is that post hoc tests are justified for each factor, if at least two factors have significant effects. It is not appropriate to do post hoc tests when no significant interactions are found. This is misleading as it causes the reader to draw erroneous conclusions. For example, in Table 1, the authors found a significant effect of stress on pHa, but no effect of sex and further no interaction between sex and stress, and so the only conclusion that can be made is that neonatal pups in general have higher levels of pHa than control animals, independent of sex. The presence of a symbol, however, now causes one to draw the erroneous conclusion that stressed males have significantly higher levels of pHa than control males but the difference between stressed and control females is not significantly, even though this difference is 0.8 compared to a difference of 0.9 between the males. The same is true in the Figures mentioned. Also, if the written description of the results is stated correctly, why use symbols that are misleading on the figures, that could lead to erroneous conclusions.” This must be corrected.

RESPONSE: Thank you for clarifying this issue. All symbols indicating differences that were not supported by factorial interactions have been remove, as requested.

2. Your rebuttal to comment 2 of reviewer 2 (interrupted breathing) could be included in the manuscript as other readers may miss this point.

RESPONSE: This comment was added to the results section, as suggested.

3. Although additional experiments are not required, it is necessary for you to discuss the possibility that hormones other than testosterone could be involved and your conclusions should be amended to reflect that you have correlations and not necessarily causality.

RESPONSE: Statement about the potential involvement of other hormones and a rational for focusing on testosterone appear in the 2nd paragraph of the discussion. The fact that our conclusion is supported by correlative evidence is mentioned in the last sentence before the section on “Perspectives” (Page 26).

Minor suggestions for revision:

1. Page 1, line 25, change factor to factors. RESPONSE: This typo has been corrected.

2. Table 1, column 7, change sex effet to sex effect. RESPONSE: This typo has been corrected.

-

3. Figure 2A, consider changing Stimulation effect to Stimulation # effect to be consistent.

RESPONSE: The suggested change has been made.

4. Figure 6A, it would be nice to use the same x-axis scale as 6B, it causes less confusion when comparing the two graphs

RESPONSE: The suggested change has been made.

5. Line 456 ...This effect was not observed in females... The sentences previously regarding the males was referring to the frequency. Are the sentences regarding the females referring to the frequency or amplitude? Please clarify.

RESPONSE: This sentence has been revised.
